# Cognitive consequences of sport-related traumatic brain injuries in adolescents in Slovakia

**DOI:** 10.1093/eurpub/ckac131.420

**Published:** 2022-10-25

**Authors:** D Hažerová, B Kažičková, M Špajdel, S Puteková, J Martinková, G Doktorová, P Sivčo, PT Pham, J Peráčková, P Peráček

**Affiliations:** 1 Department of Public Health, Faculty of Health Care and Social Work, Trnava University in Trnava, Trnava, Slovakia; 2 Department of Psychology, Faculty of Philosophy and Arts, Trnava University in Trnava, Trnava, Slovakia; 3 Department of Nursing, Faculty of Health Care and Social Work, Trnava University in Trnava, Trnava, Slovakia; 4 Department of Sports Educology and Humanistic, Faculty of Physical Education and Sports, Comenius University, Bratislava, Slovakia; 5 Department of Sports Game, Faculty of Physical Education and Sports, Comenius University, Bratislava, Slovakia

## Abstract

**Background:**

Many sports are associated with an increased risk of traumatic brain injury (TBI), often in a form of repeated minor trauma. While the pathophysiological changes of the brain after TBI have been relatively well studied, data on cognitive aspects are relatively scarce. The main objective of this study was to measure the cognitive consequences of repeated heading in a controlled set of training exercises in 21 years old football players.

**Methods:**

The study design is quasi-experiment. Participants consisted of male football players (N = 27) and were tested under 3 conditions: before the sports activity, after the sports activity not involving heading, after the sport activity focused on heading. To monitor the cognitive changes we used neuropsychological methods: the Trail Making Test (TMT), the Verbal reproduction test. Blood samples were taken to analyse the presence of biomarkers (glucose). Linear regression and repeated-measures ANOVA were used for statistical analysis.

**Results:**

The data showed significant relationships between the glucose level (before and 1-hour after the heading) and the TMT (part B) score /(F(1,22)=6.03; p=.001; R-square=.223/. Based on TMT (part B) scores, the cognitive flexibility and glucose lowered after the sessions. For both parts of TMT we found significantly worse scores after both training sessions compared to baseline testing (Part A: F(2,46)=189.354; p<.001; Eta2=.892; Part B: F(2,46)=10.191; p<.001; Eta2=.307). Post-hoc tests revealed slightly worse results in the TMT (part A) after non-heading than after the heading training which means, that the focused attention was affected. In the TMT (part B) no difference was found between the results after non-heading and heading training.

**Conclusions:**

This study has a unique potential to highlight the relations between biomarkers and psychological abilities and their possible changes caused by heading, which may have beneficial as well as damaging impact on the body and cognitive functioning.

**Key messages:**

• The findings of this study suggest a potential relationship between repeated minor head trauma and cognitive performance in young adults.

• Besides physiological changes, cognitive impact on cognitive performance may be a consequence of repeated minor head trauma; further study is required to elucidate these associations.

